# Special problems of cost analysis and cost-benefit analysis as applied to large multi-test analysers

**DOI:** 10.1155/S1463924683000206

**Published:** 1983

**Authors:** John E. Barclay

**Affiliations:** Compagnie Technicon Bureau des Garges 39 boulevard de la Muette Garges-les-Gonesse 95140 France


Journal of Automatic Chemistry, Volume 5, Number 2 (April–June 1983), pages 71–74

### Special problems of cost analysis and cost-benefit analysis as applied to large multi-test analysers

**John E. Barclay**

*Compagnie Technicon, Bureau des Garges, 39 boulevard de la Muette, 95140 Garges-les-Gonesse, France*

### Introduction

Laboratories are complex work-places with secretarial and administrative, as well as analytical, functions. They are sometimes described as factories, converting the raw material of patient specimens into the finished product of patient reports. Although this is an inexact comparison, it does suggest that management practices from manufacturing industries might be applicable to laboratories. Cost accounting is one such management practice: costs are analysed into a number of elements and these are recombined in various ways according to the purpose to be served. Such a purpose might be selection of an operating procedure, control of expenditure, or the setting of prices for the product.

In this paper, cost analysis and cost-benefit analysis for guidance or selection of operating procedures, and for controlling expenditures, will be discussed. Price setting, although it is needed by commercial laboratories and by clinical laboratories where laboratory costs are reimbursed by insurers or other third parties, is best developed using another approach (absorption costing), which is discussed in relevant textbooks [1 and 2]. Selection of operating procedures may include investment decisions for new equipment or restaffing.

Since costs are recombined in various ways to suit various purposes, it is clear that there is no unique, true and wholly accurate total cost for any particular laboratory activity. The total cost depends on the allocation of indirect labour, materials and overheads to identified direct labour and material costs.

It should also be clear that there can be no immediate single solution to the problem of running a laboratory both efficiently *and* cost-effectively.

Laboratories in a given size of hospital might be expected to have similar analytical tasks. In individual laboratories, however, the existing structure and organization in each of the domains of instrumentation, data-processing and management, results from a mixture of functional requirements and specific local historical and geographical factors:

(1) The nature and number of analyses to be performed.
(2) The medical specialities served by the laboratory.
(3) The age structure of the population.
(4) The geographical territory served.
(5) Hospital layout (tower block, remote or satellite laboratories etc.).
(6) Past instrument acquisitions.
(7) Personal interests and organization philosophy of laboratory directors, past and present.
(8) Availability of trained staff.
(9) Capital-investment policies.

Additional factors, such as the provision of specialist assay facilities for regional immunoassay or quality-control services, can lead to staffing or data-processing structures otherwise not appropriate to the main, routine work-load.

For these reasons it is difficult to define a single form of laboratory organization and equipment level as being an economic ideal under all circumstances. Cost analysis, however, allows each laboratory to use its existing equipment efficiently.

So a laboratory may be very efficient in its use of serviceable, but outdated, equipment and become cost-effective in absolute terms only after major investment in new instrumentation and organization. Consider the case of a laboratory evolving from the era of a number of single work-stations, with a central computer for data acquisition and collation, and where this computer is saturated. In short, to obtain optimal cost-effectiveness, optimal growth is needed in the domains of instrumentation, data processing and management of resources, both material and human (figure 1).

The relative importance of each domain depends on the way in which the laboratory is organized. The laboratory using multiple single work-stations, especially if it is adequately staffed, probably needs modern electronic data-processing, (EDP) to be cost-effective. As mentioned in a previous paper in this Symposium, EDP is highly desirable for the introduction of cost-analysis procedures. It is not essential, however, and lack of EDP facilities should not deter the application of cost analysis. Several benefits can be derived from the introduction of the mechanical steps of record-keeping, of checking labour time and reviewing invoices: for example improved efficiency, reduced wastage (instrument start-up and shut-down procedures are followed more closely), and identification of invoice errors [4]. In a similar way, laboratories considering computerization frequently undergo transformation as a result of documenting their activities.

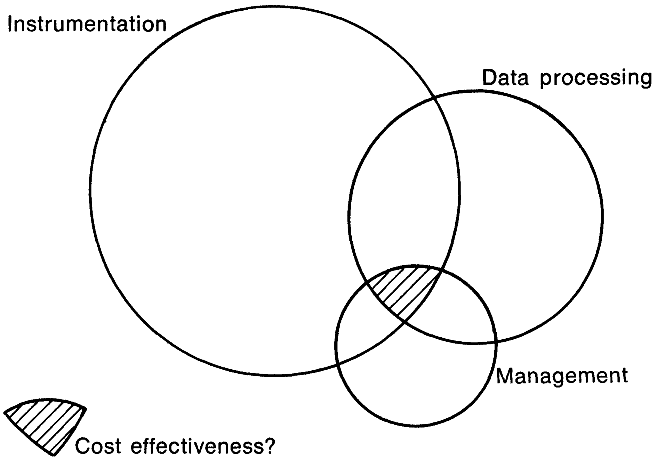

*Figure 1. Laboratory domains.*

### Large multi-test analysers

Large analysers pose several problems to cost analysis, especially when comparing a possible aquisition to currently installed equipment. Multi-test analysers may dictate that the laboratory be organized around them, especially for sample identification or special sample container requirements and result reporting. They may also have different analytical capacities, both in rate and range of analyses, as well as specific software features. Such functional detail must be studied to avoid misinterpretation of costs, both of direct operating costs and especially of the indirect costs and of costs shared between tests. Establishing comparability is the main difficulty when considering the purchase or replacement of a large multi-test analyser.

There are three basic ways of dealing with the main work-load in a laboratory, each with organizational implications (figure 2). The costing and cost-allocation process for individual tests should include the entire organization around the analyser and any complementary small analysers necessary to complete the range of tests or to increase capacity to comparable levels, including Stat or emergency testing.

### Labour costs for sample analysis

In figure 2, Situation 1 direct labour includes time spent on unrequested tests. In both Situations 1 and 2, direct labour may include such data-processing tasks as patient data entry, demographic data and comments on the sample, where these are entered into the analyser's own computer, and not a central laboratory EDP system. In Situation 2, tests requested or selectivity data must be entered. In a type 3 Situation, for example where the laboratory is organized around centrifugal analysers, these activities might be assessed as indirect labour if secretarial or EDP staff prepare worksheets, or as direct labour if carried out by technical staff at the individual work-stations. Direct labour in Situation 3 consists mainly of sample preparation, rotor filling and the transfer of rotors between instruments to enable organ profiles to be generated.

There are *qualitative* differences in labour associated with instruments and organization. Computer download capability on a large multi-test analyser would free the skilled staff from a considerable amount of clerical work—it could be carried out by secretarial or EDP personnel. The opportunity to restructure and redeploy staff in an alternative organization can be used to save costs (if there are major salary differentials) or to increase revenues or utility by undertaking new tests or activities. Time may become available for training or to cover for leaves of absence or sickness.

The savings of restructuring, or rather the cost of not restructuring, correspond to the *opportunity cost* sometimes used in setting priorities in commercial developments. Opportunity cost is the revenue lost by exploiting (or not exploiting) an alternative use of money or materials.

In commercial laboratories it is easier to identify opportunity costs than in the hospital environment where there are no revenues, it is more common to identify research activities, new tests which can be put into the 'routine' service, or to identify staffing and management problems eased by labour-saving automation.

Situation 1 — Single work-station
Sequential multiparametric analyser

Situation 2 — Single work-station
Selective—random access and/or batch

Situation 3 — Multiple work-stations

*Figure 2. Organization of routine work-load.*

Further complications exist when considering labour costs. Although labour is a variable or semi-variable cost in most countries, in others there are legislation or reimbursement conditions, which lead to high staff/work-load ratios. In these cases, labour must be regarded as a fixed cost, and opportunity costs become more important when justifying investment.

The allocation of labour to individual tests will be dealt with later.

### Reagent and consumable costs

Reagent volume per test is frequently used to calculate cost/test in conjunction with invoice prices for reagents. This can be misleading because of priming volumes, batch size effects [3], daily reagent preparation and wastage due to short shelf-life. These factors vary considerably between instruments. Similarly, calibration and control protocols use reagents and consumables, and these are also instrument dependent. Total reagent utilization over a trial period of two weeks or more will lead to more accurate figures for comparison.

### Allocation of costs

Many elements of cost cannot be traced directly to specific tests or requests. Four cases will be considered briefly.

### *Shared direct costs*

In a multi-test analyser these are the costs of shared reagents (for example buffers or wash), consumables and labour. Division by the sum of the number of tests is adequate for instruments or groups of instruments performing tests *selectively*. For profiles which have been requested, each constituent test should be counted. When a non-selective analyser is used, each constituent test in a profile should count where profiles are requested, otherwise the *number of tests requested* for individual chemistries should be used. This distinction requires careful analysis of the laboratory work-load and request patterns.

### *The costs of quality control*

Quality-control (QC) costs should be separated out as an indirect cost rather than a shared direct cost. Cost of calibrators, precision controls, graph paper and labour involved should be identified, but reagents used for the analyses may be left in the total reagent consumption ascribed to samples over the trial period.

QC is an essential part of laboratory management and is often supervised by one individual. A QC 'work-station' can be defined and the identified costs for all instruments attributed to this. For most purposes there should be no need to make a suballocation to a multi-test instrument or to another analytical work-station.

Some large multi-test analysers have sophisticated internal QC programs. The automatic printing of charts, or of automatic assignment of values to unassayed controls, can result in a reduction of labour (secretarial and analytical). These benefits may be clearly shown in the QC function of the multi-test analyser is separated out and included in a notional QC work-station.

In calculating expected QC costs on a new analyser, the total reference serum consumption over a defined period should be used—shelf-life, package size etc.—rather than volume of serum needed per test. This avoids complex calculation of QC serum consumption by individual instruments and repeated calculation in the event of price changes.

### *Indirect costs*

Examples of indirect costs have already been given in this Symposium by Professor Haeckel. The sum total of indirect costs in the laboratory is usually much higher than the direct costs. When calculating total cost per test or per work-station, the selection of the correct method of assigning these to the identified costs is essential to avoid misinterpretation. Broughton *et al.* [4] have shown that allocation of indirect costs by the direct cost results in an excessive weighting for those tests involving expensive reagents, and for those which are labour-intensive. At any work-station, allocation by the number of *tests* performed penalizes non-selective analysers, since no additional indirect costs are involved in measuring the whole panel of tests rather than any part of it. Broughton *et al.* recommend that indirect costs should be allocated *per request.* This is rational and appealing. The resulting indirect cost/request is constant and is equivalent to a 'handling charge' by the laboratory. The direct costs of the component tests are more evident, and more easily compared with other laboratories or alternative work-load and organizational situations proposed for the same laboratory.

### *Capital costs*

A full treatment of capital costs is beyond the scope of this paper. Normally, capital expenditure plus service costs are summed and amortized over a reasonable period of time. The annual amortization for all laboratory equipment is summed and treated as an indirect cost. Real capital outlays for multi-test analysers must be identified, i.e. the purchase prices after any discount or allowance, but including any special 'starter kit' of spares. Costs of necessary modifications to the laboratory or additional equipment must also be considered: these include the usual services (electricity, drains etc.) and also air-conditioning, additional centrifuges, etc. Expenses involved in retraining staff should also be identified.

Two aspects of service costs merit special attention. Service contracts issued by manufacturers differ quite widely in terms of the number of free visits, labour charges, parts replacement, parts to be purchased and held by the laboratory. Care should be taken to establish total annual expenditures and not simply the service contract charge. Secondly, some multi-test analysers have internal on-line quality-control programmes (for example the Westgard Algorithm [5]). These programmes are automatic and only involve the operator when errors are present: by indicating that maintenance is necessary. Using QC programmes may slightly increase direct labour, but this might be offset by reduced service costs.

### Comparison of alternative instruments and organization

Although intermittent cost analysis is a useful means of controlling costs and limiting expenditure, the real benefit of cost analysis is in the simplification of the process of comparing the economics of alternative instruments and organization. For the first purpose a relatively simple approach may be adequate. Broughton *et al.* collected annual data on test volume, request volume, salaries etc.: total indirect costs are then the difference between total costs and identifiable direct costs. Their approach measures overall managerial efficiency and cost control, but it does not give enough detail for evaluating different instruments. There are three important publications giving guidelines to cost comparison. The earliest approach (Coopers-Lybrand [6]), is complex, omits overheads and is not easily applied. Krieg and Israel [7] and Worth [8] use two different approaches to compensate for work-load differences. Worth believes that if a model work-load consisting of a broad spectrum of analytes is considered (25 for instance), then combinations of instruments of different capacity can be compared. Kreig also uses a model work-load, but he standardizes labour times using the CAP unit values (which are probably not applicable in Europe). In his model, indirect costs are allocated to functional work-stations which may physically exist at instruments or be artificial. These form a 'work-station tree', with administration, and other general costs allocated to the top of the tree. An example is shown in figure 3. This approach draws attention to the non-analytical features of instruments (figure 4). A modern multi-test analyser, with software featuring on-line QC, multiple reference ranges, multiple report formats etc. is designed to produce *qualified* results in a format easily assimilated, and to guide

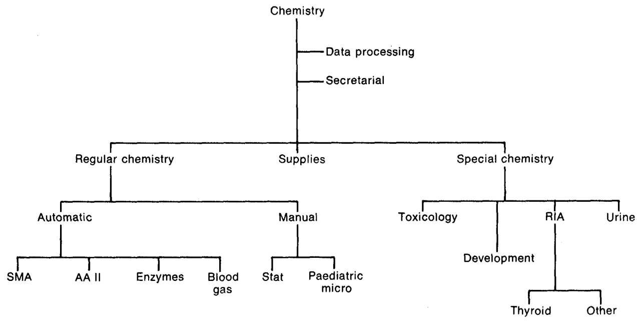

*Figure 3. Work-station tree—after Krieg and Israel* [7].

Sample identification
Test selectivity
Patient demographics
Sample comments
On-line QC
External data entry
Result review and editing
Patient data storage and retrieval
Print-out—variable format, remote printing
Printing—worksheets, labels, etc.
Appointment service, inventory control and similar software features

*Figure 4. Some non-analytical instrument features.*

physicians in their interpretation. These are features intended to improve *utility* rather than analytical cost-benefit. Their importance is greater if there is no central EDP system. An instrument with these features could partly replace secretarial or medical supervisory activities. In this case an extension of Kreig and Israel's approach is appropriate.

An attempt should be made to identify the analyser's non-analytical functions and these should be assigned to relevant work-stations (for example sample preparation, QC, secretarial). Labour costs can be then more accurately assigned.

### Cost-benefit analysis

Cost-benefit analysis is much more difficult to evaluate—many benefits are qualitative and remotely related to costs.

Economic
Convenience: for physician and laboratory
Quality } improved utility
Turn-about time } improved utility
New method introduction } improved utility
Organization
Managerial: training, promotion, redeployment of skill
Environmental: less biohazard, noise, etc.

*Figure 5. Some types of benefit for cost-benefit analysis.*

Some of the benefits obtained from multi-test analysers are presented in figure 5.

Assessment of economic benefit can be made in monetary terms—the *payback period* for example. Opportunity cost should be integrated in this calculation. Where relevant, improved clinical utility of the laboratory may be measured by a change in the number of routine requests—fewer Stat requests during normal working hours—and an increase in the number of special test requests. Managerial efficiency can be measured in terms of total annual budget, labour costs, staff turnover rate, degrees and diplomas obtained by the staff. Benefit, like beauty, lies in the eye of the beholder.

### Summary

Large multi-test analysers have a major impact on the organization of a laboratory. Cost analysis and cost-benefit analysis of such instruments must taken into account all the organizational changes, including staff structure and numbers and ancillary equipment, required around the analyser. The use of a functional work-station tree-diagram can lead to a more accurate cost allocation and help in comparisons of different instruments and organizational structures.

### References

1. Willsmore, A. W., *Accounting for Management Control* (Pitman Publishing, Bath, 1971).
2. Bennington, J. L., Boer, G. B. and Louvan, G. E., *Management and Cost Control Techniques for the Clinical Laboratory* (University Park Press, Baltimore, 1977).
3. Haeckel, R., *Medical Progress through Technology*, **3** (1975), 65.
4. Broughton, P. M. G. and Hogan, T. C., *Annals of Clinical Biochemistry*, **18** (1981), 330.
5. Westgard, J. O., Barry, P. L. and Hunt, M. R., *Clinical Chemistry*, **27** (1981), 483.
6. Coopers-Lybrand, *Procedure for Determining Test Costs in Pathology Laboratories* (DHSS, 1975).
7. Krieg, A. F. and Israel, M., *American Journal of Clinical Pathology*, **69** (1978), 525.
8. Worth, H. J., *Journal of Automatic Chemistry*, **2** (1980), 125.

### CONFERENCE ANNOUNCEMENT

*Second International Congress on Automation and New Technology in the Clinical Laboratory*

At Barcelona, Spain from 15–18 October 1984. A preliminary programme has just been issued and papers and registrations are invited; the congress will consist of symposia, workshops and poster sessions. Topics will include:

- Automation and new technology in microbiology
- Instrument evaluation
- The impact of new technology on emergency technology
- Equipment for small laboratories
- Quality control of automatic cell counting and flow cytometry
- Cellular chemistry and neurochemistry
- Impact of microcomputers on the clinical laboratory
- Automation and new technology in haematology
- Quantitative cytometry
- Nuclear magnetic resonance
- New technology in coagulation and immunology.

Languages are English, Spanish and French with simultaneous translation.

The members of the scientific board include F. L. Mitchell, R. Haeckel, T. D. Geary, M. Helm, R. Galimany, J. Bierens de Haan, P. Bonini, J. L. Gentilini, C. Burtis, S. Bauer and F. J. Gella. And M. J. Alsina, E. Esquerdo, M. Domenech, R. Galimany, F. J. Gella, C. Pastor and M. Vives make up the local organizing committee.

A trade exhibition of products and equipment related to automation and new technology will be held during the congress.

*Details from Dr R. Galimany, Secretary of the Second Congress, Apartado de Correos 543, Barcelona, Spain.*

